# Selective Regulation of Cytoskeletal Dynamics and Filopodia Formation by Teleost Leukocyte Immune-Type Receptors Differentially Contributes to Target Capture During the Phagocytic Process

**DOI:** 10.3389/fimmu.2018.01144

**Published:** 2018-06-28

**Authors:** Dustin M. E. Lillico, Joshua G. Pemberton, James L. Stafford

**Affiliations:** Department of Biological Sciences, University of Alberta, Edmonton, AB, Canada

**Keywords:** phagocytosis, innate immunity, immunoregulatory receptors, signal transduction, actin cytoskeleton, comparative immunology, teleost fish

## Abstract

Phagocytosis evolved from a fundamental nutrient acquisition mechanism in primitive unicellular amoeboids, into a dynamic and complex component of innate immunity in multicellular organisms. To better understand the cellular mechanisms contributing to phagocytic processes across vertebrates, our research has focused on characterizing the involvement of innate immune proteins originally identified in channel catfish (*Ictalurus punctatus*) called leukocyte immune-type receptors (IpLITRs). These unique teleost proteins share basic structural as well as distant phylogenetic relationships with several immunoregulatory proteins within the mammalian immunoglobulin superfamily. In the present study, we use a combination of live-cell confocal imaging and high-resolution scanning electron microscopy to further examine the classical immunoreceptor tyrosine-based activation motif (ITAM)-dependent phagocytic pathway mediated by the chimeric construct IpLITR 2.6b/IpFcRγ-L and the functionally diverse immunoreceptor tyrosine-based inhibitory motif-containing receptor IpLITR 1.1b. Results demonstrate that IpLITR 1.1b-expressing cells can uniquely generate actin-dense filopodia-like protrusions during the early stages of extracellular target interactions. In addition, we observed that these structures retract after contacting extracellular targets to secure captured microspheres on the cell surface. This activity was often followed by the generation of robust secondary waves of actin polymerization leading to the formation of stabilized phagocytic cups. At depressed temperatures of 27°C, IpLITR 2.6b/IpFcRγ-L-mediated phagocytosis was completely blocked, whereas IpLITR 1.1b-expressing cells continued to generate dynamic actin-dense filopodia at this lower temperature. Overall, these results provide new support for the hypothesis that IpLITR 1.1b, but not IpLITR 2.6b/IpFcRγ-L, directly triggers filopodia formation when expressed in representative myeloid cells. This also offers new information regarding the directed ability of immunoregulatory receptor-types to initiate dynamic membrane structures and provides insights into an alternative ITAM-independent target capture pathway that is functionally distinct from the classical phagocytic pathways.

## Introduction

Phagocytosis is a vital innate immune response that involves the engulfment, destruction, and removal of extracellular targets; such as microbes, necrotic or apoptotic cells, and cellular debris (1). The unique ability of phagocytic cells to recognize and engulf large particulate targets depends on the surface expression of specialized immunoregulatory receptors. Well known mammalian phagocytic receptor-types include complement receptors (1–4), members of the Fc receptor (FcR) family (1–3), and dectin-1 (5). Studies using these model immune proteins have shown that phagocytosis is a multifaceted process that tightly regulates the active capture, ingestion, and subsequent destruction of various microbial targets (1–5). Phagocytic receptors relay their interactions with extracellular targets into dynamic filamentous (F)-actin remodeling events that reshape the plasma membrane through specialized intracellular signaling events (1, 6–9). Generally, each of these phagocytic pathways requires localized phospholipid metabolism and the engagement of actin nucleation and regulatory factors that link surface receptor activation with the cytoskeletal machinery to facilitate target engulfment (1, 6–9).

Interactions between phagocytic receptors and extracellular targets are not always reliant on passive binding events; rather, phagocytes actively increase the incidence of target-binding events through the formation of unique finger-like extensions of the plasma membrane called filopodia (10–13). These membrane protrusions are composed of un-branched filaments of polymerized F-actin that vary greatly in length (1–100 µm), thickness (0.1–0.3 µm), molecular composition, and geometry (10–13). Early studies using scanning electron microscopy (SEM) revealed that mouse peritoneal macrophages formed cord-like extensions that arose from the plasma membrane to tether extracellular targets to the cell surface (14). The formation of filopodia following bacterial lipopolysaccharide stimulation of macrophages was also shown to occur through the phosphorylation of various intracellular signaling mediators (15, 16). Following the initial contact with extracellular targets, filopodia quickly retract back toward the cell body, resulting in the immobilization and tethering of targets to the plasma membrane (12–15, 17, 18). This action allows for additional surface receptor–target interactions to occur that reinforce the transduction events responsible for the temporal activation of the phagocytic process (12, 13, 18). While the ability of phagocytes to actively deploy filopodia has been demonstrated, relatively little is known about the specific intracellular molecules and receptor-types that participate in filopodial dynamics within innate immune cells. Across eukaryotes, the formation of filopodia during diverse cellular events has been reported to require several classes of protein kinases and kinase-associated molecular scaffolds, small Rho-family GTPases [e.g., Ras-related C3 botulinum toxin substrate 1/2 (Rac1/2) and cell division control protein 42 homolog (Cdc42)], various cytoskeletal elements including components of the actin polymerization machinery (e.g., myosins and formins), as well as the generation of membrane-embedded phosphatidylinositol 3,4,5-trisphosphate (PtdIns(3,4,5)P_3_) (1, 7, 17–22). While some of the molecular constituents required for filopodia formation in mammalian macrophages have been described, much less is known about what specific cell-surface receptor-types trigger the formation of these structures and whether the ability of specific immunoregulatory receptor-types that control membrane dynamics are conserved in basal vertebrates.

Our research has progressively established channel catfish (*Ictalurus punctatus*) leukocyte immune-type receptors (IpLITRs) as a unique comparative model to understand mechanisms of immunoregulatory receptor-mediated control of innate cellular immunity in vertebrates. IpLITR-types are co-expressed by catfish myeloid and lymphoid cells (23, 24) and sub-types of these receptors have also recently been shown to serve as surface markers on subsets of cytotoxic T cells during viral infection in catfish (25). To date, IpLITRs have only been reported in the channel catfish (23, 24) and zebrafish (*Danio rerio)* (24, 26, 27) but they are likely not exclusive to these species as we have identified IpLITR-related sequences in several other fish species from the non-redundant protein sequence databases (*unpublished data*). Overall, IpLITRs share basic structural as well as distant phylogenetic relationships with several immunoregulatory proteins within the mammalian immunoglobulin superfamily (23) and they appear to be important regulators of innate cellular responses *via* classical as well as unique biochemical signaling networks (28–30). Although our functional characterization of IpLITR-types has relied on heterologous expression of teleost proteins in mammalian cells, this strategy has allowed us to demonstrate important conserved aspects regarding IpLITR-mediated immunoregulatory signaling events and revealed some unanticipated aspects regarding the versatility of IpLITR-mediated transduction. In particular, when expressed in the mammalian myeloid rat basophilic leukemia (RBL)-2H3 cell line, we have previously shown that IpLITR 2.6b/IpFcRγ-L activates phagocytosis using a characteristic intracellular transduction response that is reminiscent of the prototypical mammalian immunoreceptor tyrosine-based activation motif (ITAM)-dependent FcR phagocytic pathway (29). Subsequently, we also described an alternative phagocytic mechanism mediated by an immunoreceptor tyrosine-based inhibitory motif (ITIM)-containing receptor called IpLITR 1.1b. This unique IpLITR 1.1b-mediated mechanism exhibited reduced target engulfment, overall; but, alternatively, this receptor sub-type featured a significantly enhanced ability to capture extracellular beads (29). While the atypical pathway requires active engagement of the actin polymerization machinery, IpLITR 1.1b-expressing cells were insensitive to pharmacological inhibitors that blocked the classic signaling components of ITAM-dependent phagocytosis. Furthermore, the ability of IpLITR 1.1b-expressing RBL-2H3 cells to capture beads was not affected at 27°C, an incubation temperature that completely inhibited IpLITR 2.6b/IpFcRγ-L phagocytosis (29). Imaging studies of fixed IpLITR 1.1b-expressing cells showed that the presence of this receptor produced extended plasma membrane structures that appeared to participate in the capture and tethering of microsphere targets to the cell surface; a phagocytic phenotype that was not observed fpr IpLITR 2.6b/IpFcRγ-L-expressing cells during the phagocytic response (29). However, whether or not IpLITR 1.1b was directly controlling the formation of filopodia and how these structures were utilized for target interactions could not be deciphered from fixed cells.

In the present study, we utilized a combination of live-cell imaging (LCI) and high-resolution SEM to provide detailed new information regarding IpLITR-induced plasma membrane dynamics during the phagocytic process as well as explore the potential for receptor-selective filopodia formation. Our results support the hypothesis that F-actin-dense protrusions are indeed produced by IpLITR 1.1b-expressing cells. In addition, we show that during the early stages of the IpLITR 1.1b-mediated phagocytic process these filopodia-like structures often retract after target contact to secure the captured microspheres to the cell surface. This unique target-capturing phenotype is followed by the formation of phagocytic cup-like structures at the membrane interface and, in some cases, the eventual engulfment of the immobilized microspheres. At the reduced incubation temperature of 27°C we also show that although the membrane structures had repressed mobility, dynamic filopodia structures were still generated by IpLITR 1.1b-expressing cells, which continued to facilitate sustained cell–target interactions. Conversely, no F-actin dynamics or any associated membrane activity was seen in IpLITR 2.6b/IpFcRγ-L-expressing cells; likely due to an inability of this receptor to promote or maintain F-actin polymerization events below 37°C. Overall, results from these studies show that a unique IpLITR sub-type can selectively regulate filopodia formation over a range of incubation temperatures. This also reinforces the use of IpLITRs as an alternative vertebrate model for investigating the integration of immune cell membrane and cytoskeletal dynamics during the coordinate control of the phagocytic process.

## Materials and Methods

### Generation of Stable IpLITR-Expressing RBL-2H3 Cells

The transfection and selection of RBL-2H3 cells stably expressing N-terminal hemagglutinin (HA)-tagged IpLITRs in the pDisplay vector was performed as described (29, 30). IpLITR 2.6b/FcRγ-L is a chimeric receptor, which contains two extracellular Ig-like domains (GenBank Accession: ABI23577) fused with the ITAM-containing cytoplasmic tail (CYT) region of the signaling adaptor IpFcRγ-L. This chimeric receptor construct was used to examine ITAM-mediated responses transmitted by the teleost adaptor IpFcRγ-L (30, 31). IpLITR 1.1b (GenBank Accession: ABI16050) encodes the full length TS32.17 L1.1b sequence and contains four extracellular Ig-like domains and a six tyrosine-containing CYT. Transfected RBL-2H3 cells were grown at 37°C and 5% CO_2_ in complete culture media [minimal essential media (MEM); Sigma-Aldrich, St. Louis, MO, USA] supplemented with Earl’s balance salt solution (GE Healthcare, Baie d’Urfe, QC, Canada), 2 mM l-Glutamine (Life Technologies, Inc., Burlington, ON, Canada), 100 U/mL penicillin (Life Technologies, Inc.), 100 µg/mL streptomycin (Life Technologies, Inc.), 400 µg/mL G418 disulfate salt solution (Sigma-Aldrich, St. Louis, MO, USA), and 10% heat inactivated fetal bovine serum (Sigma-Aldrich). Surface expression of IpLITRs was monitored by flow cytometry using an αHA monoclonal antibody (mAb; Cedarlane Laboratories Ltd., Burlington, ON, Canada) as described previously (29, 30).

### SEM of IpLITR-Mediated Phagocytosis

Scanning electron microscopy of IpLITR-mediated phagocytosis was performed using antibody-opsonized 4.5-µm microsphere targets. Briefly, 3 × 10^5^ RBL-2H3 cells expressing either IpLITR 2.6b/IpFcRγ-L or IpLITR 1.1b were plated onto a sterile 18-mm diameter #1 1/2 circular coverslip (Electron Microscopy Sciences, Hatfield, PA, USA) and cultured overnight at 37°C with 5% CO_2_ in a six-well tissue culture plate (Fisher Scientific Company, Ottawa, ON, Canada). The following day cells were washed with phosphate buffered saline (PBS) and then incubated in phagocytosis buffer (1:1 mixture of 1× PBS containing 2 mg/mL bovine serum albumin, Sigma-Aldrich) and 1× Opti-MEM reduced serum medium (Fisher Scientific Company) containing 9 × 10^5^ 4.5-µm target microspheres (beads; Polybead^®^ Carboxylate YG microspheres; Polysciences, Warrington, PA, USA) opsonized with 10 µg/mL of αHA mAb (Cedarlane Laboratories Ltd., Burlington, ON, Canada) or 10 µg/mL of the isotype control mouse IgG3 (Beckman Coulter, Mississauga, ON, Canada). Antibody opsonization was performed by absorbing them onto protein A precoated microspheres (isolated from *Staphylococcus aureus*; Sigma-Aldrich) as previously described (28, 29). Plates containing cells and target beads were then centrifuged at 1,500 rpm for 1 min to synchronize cell–bead interactions and then incubated for 1 h at either 27 or 37°C. For some experiments, the IpLITR-expressing cells were pre-treated for 1 h with 12.5 µM of the F-actin inhibitor Latrunculin B (EMD Millipore; Burlington, MA, USA) prior to their incubation with opsonized beads. Cells then were fixed with 2.5% glutaraldehyde/2% paraformaldehyde in a 0.1 M phosphate buffer solution. Dehydration of cells was then performed by sequential treatments with ethanol and hexamethyldisilazane according to previously described procedures (32, 33). After dehydration, coverslips were mounted onto round metal double-sided sticky stubs and coated with an ultrathin coating of gold/plutonium *via* a Hummer 6.2 Sputter Coater (Anatech USA, Hayward, CA, USA) and then imaged using a Philips/FEI XL30 SEM microscope (FEI: Hillsboro, OR, USA). Image analysis was performed using the Scandium 5.0 software (Emsis: Muenster, Germany).

### Generation of Stable LifeAct-GFP-Expressing RBL-2H3 Cells

Cytoskletal dynamics within IpLITR-expressing RBL-2H3 stable cell lines were examined using LifeAct-GFP (a generous gift from Dr. Nicholas Touret, University of Alberta), which is a C-terminal conjugated green fluorescent protein (GFP) probe that binds specifically to F-actin molecules (34). IpLITR-expressing RBL-2H3 cells were stably transfected with LifeAct-GFP using nucleofection (Amaxa Cell Line Nucleofector Kit T, RBL-2H3; Lonza, Cologne, Germany) according to the manufacture’s recommended protocol. Briefly, IpLITR 2.6b/IpFcRγ-L- and IpLITR 1.1b-expressing RBL-2H3 cells were grown to confluence (~2.6 × 10^6^ cells) in a six-well tissue culture plate, harvested, and then washed with PBS. Cells were then mixed with 100 µL of Cell Line Nucleofector Solution T (Amaxa) and 5 µg of LifeAct-GFP plasmid. Samples were transferred into a nucleofection cuvette and transfected using the Nucleofector II Device (Amaxa) using the program designated for the RBL-2H3 cell line (Program X-001). Cells were then placed into pre-warmed selection media (complete MEM supplemented with 400 µg of G418) and incubated at 37°C with 5% CO_2_ until confluent. Cells were then harvested and the GFP positive cells were sorted using a FACSCanto II (BD Bioscience). The GFP positive cells were then plated and incubated until they grew to confluence prior to being stained for IpLITR expression to verify that co-expression of LifeAct-GFP did not alter IpLITR surface expression levels.

### LCI of IpLITR-Mediated Phagocytosis

Rat basophilic leukemia-2H3 cells stably co-expressing IpLITR and LifeAct-GFP (3 × 10^5^ cells) were plated onto 50 mm μ-dishes (Ibidi; Madison, WI, USA) the day prior to imaging. The following day, cells were washed with PBS and then incubated in phagocytosis buffer containing either 9 × 10^5^ αHA mAb-opsonized 4.5 µm non-fluorescent beads or αHA mAb-opsonized 4.5 µm blue beads (BB) (Fluoresbrite™ Carboxy BB microspheres; Polysciences, Warrington, PA, USA) and placed into a microscope stage chamber, which was supplied with 5% CO_2_ and heated to 37 or 27°C. Immediately after the addition of target beads, images were collected at 10-s intervals for ~8 min using a Zeiss LSM 710 laser scanning confocal microscope (objective 60×, 1.3 oil plan-Apochromat; Munich, Germany) located at the Cross Cancer Institute Microscopy Facility (Faulty of Medicine & Dentistry; University of Alberta). Imaging data were analyzed using the Zen software package (2011; Carl Zeiss; Oberkochen, Germany) and ImageJ (ImageJ version 1.51p; Rasband, 1997–2017).

## Results

### IpLITR 2.6b/IpFcRγ-L-Mediated Phagocytosis

**Figure 1:**
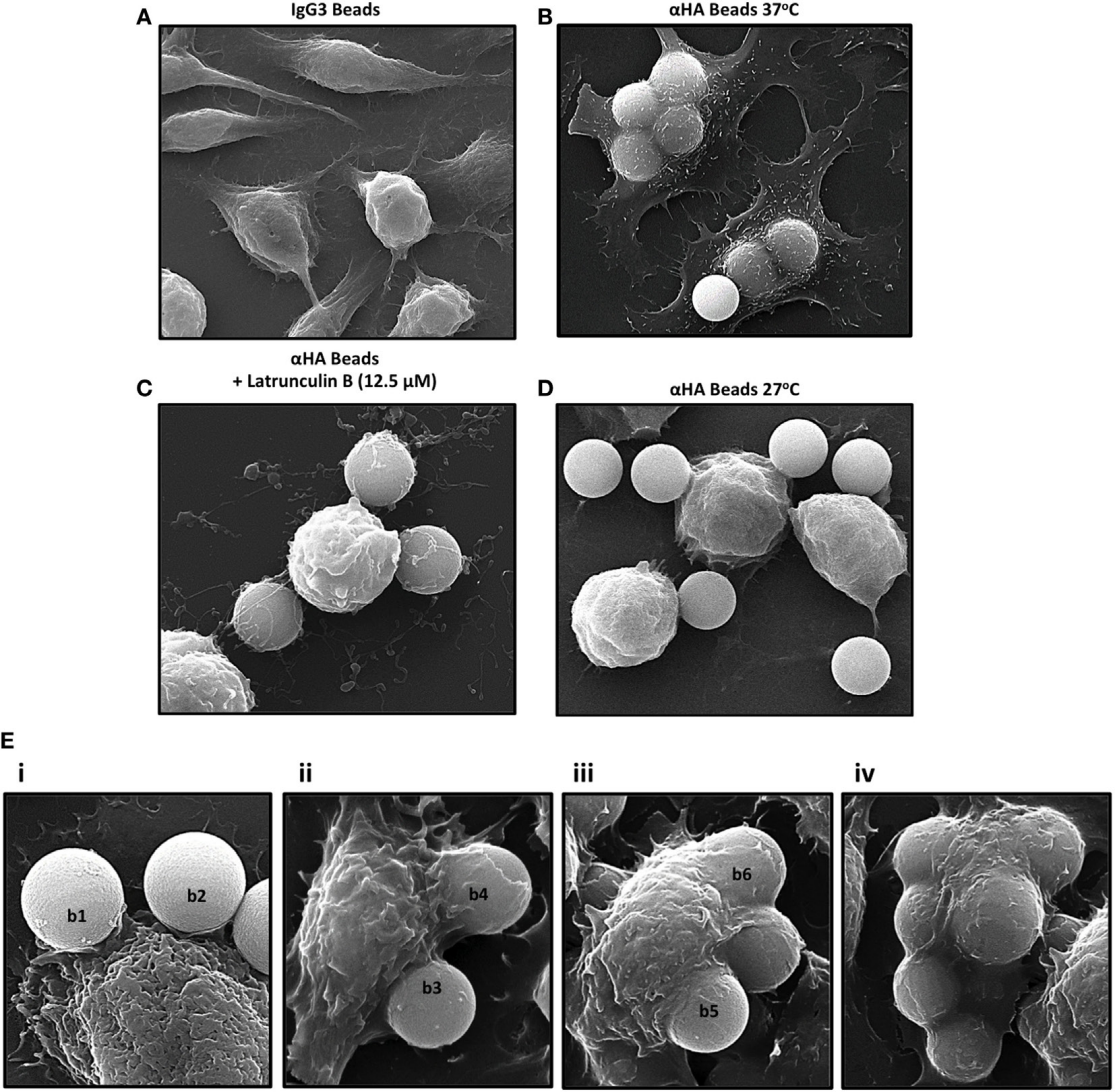
Scanning electron microscopy (SEM) of IpLITR 2.6b/IpFcRγ-L-mediated phagocytosis. IpLITR 2.6b/IpFcRγ-L-expressing rat basophilic leukemia-2H3 cells (3 × 10^5^) were incubated at 37°C for 1 h with 9 × 10^5^ IgG3-coated 4.5 µm microspheres **(A)** or with 9 × 10^5^ αHA monoclonal antibody (mAb)-coated 4.5 µm microspheres **(B)** prior to imaging using a Philips/FEI XL30 SEM microscope (FEI: Hillsboro, OR, USA). Cells (3 × 10^5^) were also pretreated for 1 h with 12.5 µM of the F-actin inhibitor Latrunculin B prior to their incubation with 9 × 10^5^ αHA mAb-coated 4.5 µm microspheres **(C)** or incubated at 27°C for 1 h with 9 × 10^5^ αHA mAb-coated 4.5 µm microspheres **(D)** prior to imaging. IpLITR 2.6b/IpFcRγ-L-expressing cells (3 × 10^5^) were also incubated at 37°C for various times (e.g., 4, 8, 16, and 32 min) with 9 × 10^5^ αHA mAb-coated 4.5 µm microspheres and representative SEM images of the progressive stages of IpLITR 2.6b/IpFcRγ-L/IpFcRγ-L-mediated phagocytosis are shown in panels i–iv **(E)**. Specific beads are labeled (b1–b5) as described in the results section.

When incubated with isotype control IgG3 beads, no internalized or surface-bound targets were observed (Figure 1A). Comparatively, when incubated with αHA-opsonized beads (1 h at 37°C), IpLITR 2.6b/IpFcRγ-L-expressing RBL-2H3 cells displayed a characteristic flattened morphology and had multiple internalized beads with few surface-bound targets (Figure 1B). Pretreatment of the cells with a selective inhibitor of actin polymerization, Latrunculin B, abrogated bead internalization; although many of the beads remained associated with the cell surface (Figure 1C). Not surprisingly, cells treated with Latrunculin B also had a rounded morphology (compare Figure 1B with Figure 1C). When IpLITR 2.6b/IpFcRγ-L-expressing cells were incubated with target beads for 1 h at the reduced temperature of 27°C to inhibit phagocytosis, engulfment was indeed abrogated but the target microspheres appeared to be loosely associated with the plasma membrane (Figure 1D). Notably, when IpLITR 2.6b/IpFcRγ-L-expressing cells were incubated at 27°C they also had an overall rounded appearance. To capture additional representative images during the early stages of the IpLITR 2.6b/IpFcRγ-L phagocytic process, SEM was performed using cells incubated with the αHA-opsonized beads for shorter time periods at 37°C (i.e., 4, 8, 16, and 32 min). Characteristic stages of IpLITR 2.6b/IpFcRγ-L phagocytosis, beginning with cell-bead contacts through to complete target internalization are shown in Figure 1E (panels i-iv). During IpLITR 2.6b/IpFcRγ-L-mediated phagocytosis, phagocytic cup formation (Figure 1E; i, beads b1 and b2) occurs after initial contact of the target beads with the cell membrane. The cup progresses as extended pseudopod-like structures (Figure 1E; ii, b3 and b4) around the outer edges of the beads, which then continues over the beads (Figure 1E; iii, b5 and b6) until the targets are internalized (Figure 1E; iv).

### IpLITR 2.6b/IpFcRγ-L-Mediated Phagocytosis at Different Temperatures

Scanning electron microscopy provided high-resolution static images of the IpLITR 2.6b/IpFcRγ-L-mediated phagocytic process. However, to observe the dynamic membrane remodeling events that occur during cell–target interactions required the use of real-time LCI. To achieve this, IpLITR 2.6b/IpFcRγ-L-expressing RBL-2H3 cells stably expressing the fluorescent probe LifeAct-GFP were used. This allowed us to visualize and track distinct F-actin polymerization events (green) and associated membrane dynamics that occur starting from initial target contacts through to the engulfment of individual microspheres. Importantly, stable expression of LifeAct-GFP did not reduce the surface expression of IpLITRs (Figure S1 in Presentation 1 of Supplementary Material).

**Figure 2:**
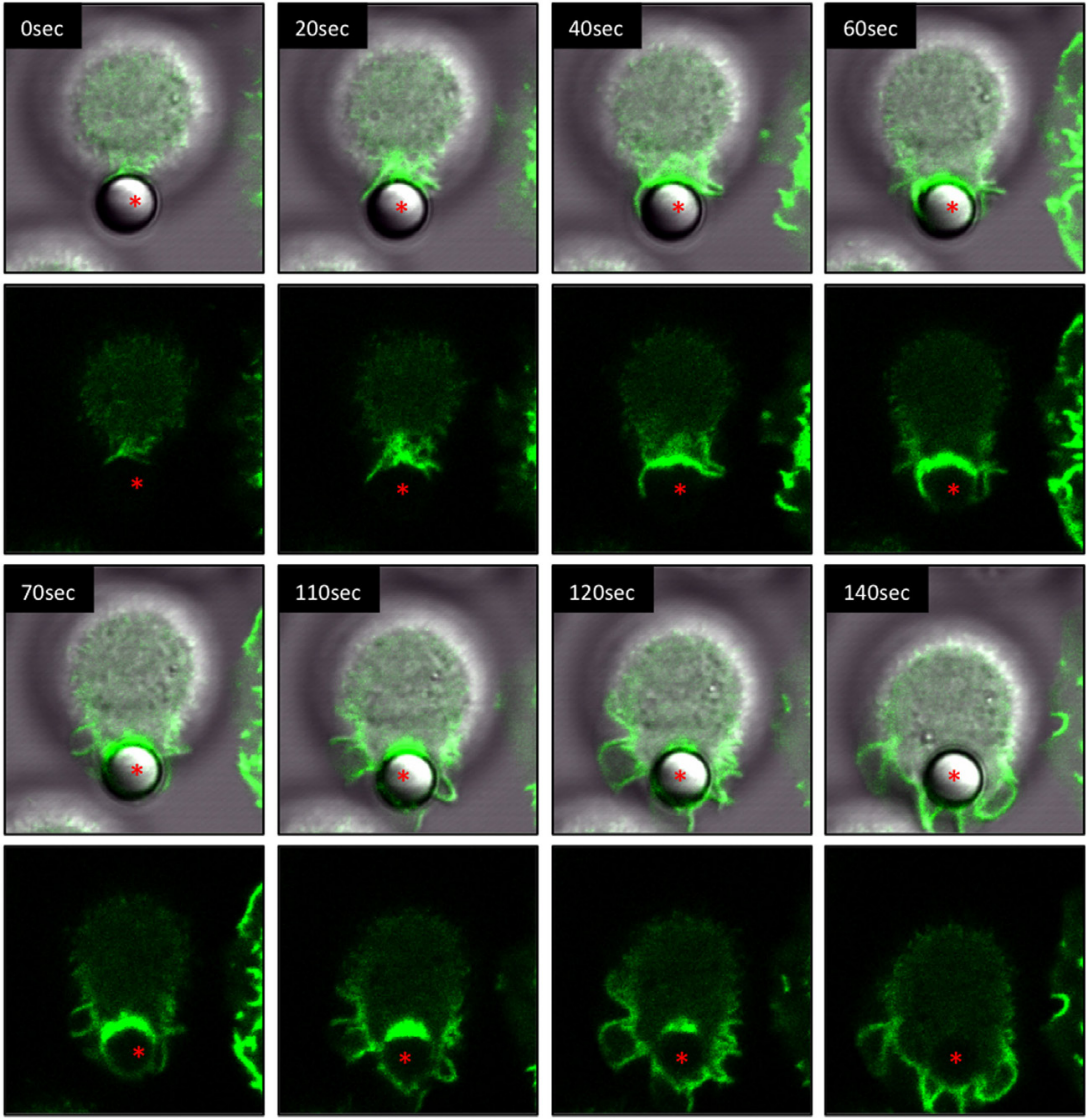
Live-cell imaging of IpLITR 2.6b/IpFcRγ-L-mediated phagocytosis. Rat basophilic leukemia-2H3 cells (3 × 10^5^) stably co-expressing IpLITR 2.6b/IpFcRγ-L and LifeAct-GFP were incubated at 37°C with 9 × 10^5^ αHA monoclonal antibody-coated 4.5 µm microspheres. Immediately after the addition of target beads, images were collected at 10 s intervals for ~8 min using a Zeiss LSM 710 laser scanning confocal microscope (objective 60×, 1.3 oil plan-Apochromat; Munich, Germany). Both the brightfield-LifeAct-GFP merged views (top panels) and the LifeAct-GFP views alone (bottom panels) are shown with the location of the target microsphere indicated with an asterisk. Representative time-stamps were extracted from Video S1 in Presentation 2 of Supplementary Material.

IpLITR 2.6b/IpFcRγ-L mediates the internalization of αHA-opsonized target beads through a series of distinctive phases of F-actin dependent plasma membrane remodeling events. These F-actin polymerization dynamics are shown in a representative LCI time-lapse video (Video S1 in Presentation 2 of Supplementary Material) and in an associated series of time-stamped static images extracted from the LCI video (Figure 2). In Video S1 in Presentation 2 of Supplementary Material, both brightfield images merged with LifeAct-GFP (S1a) as well as the LifeAct-GFP signal alone (S1b) are displayed with the non-fluorescent microspheres clearly visible in the brightfield panels. For the time-stamps, both the brightfield-LifeAct-GFP merged views (top panels) and the LifeAct-GFP views alone (bottom panels) are shown with the location of the target microsphere indicated with a red asterisk. During the initial stages of IpLITR 2.6b/IpFcRγ-L-mediated phagocytosis, actin polymerization (green) is clearly visible at the cell surface-target interface in what appears as a phagocytic cup-like structure (Figure 2, asterisk, 0–40 s). As the phagocytic process proceeds, polymerization of F-actin is visible along the leading edges of extended pseudopods (Figure 2; 40–70 s). The accumulated F-actin behind the bead then depolymerizes as the pseudopods seal together and the microsphere sinks into the cell (Figure 2; 120–140 s). Another representative LCI time-lapse video showing IpLITR 2.6b/IpFcRγ-L-mediated phagocytic behavior is provided in Video S2 in Presentation 2 of Supplementary Material with the associated time-stamped static images provided in Figure S2 in Presentation 1 of Supplementary Material.

**Figure 3:**
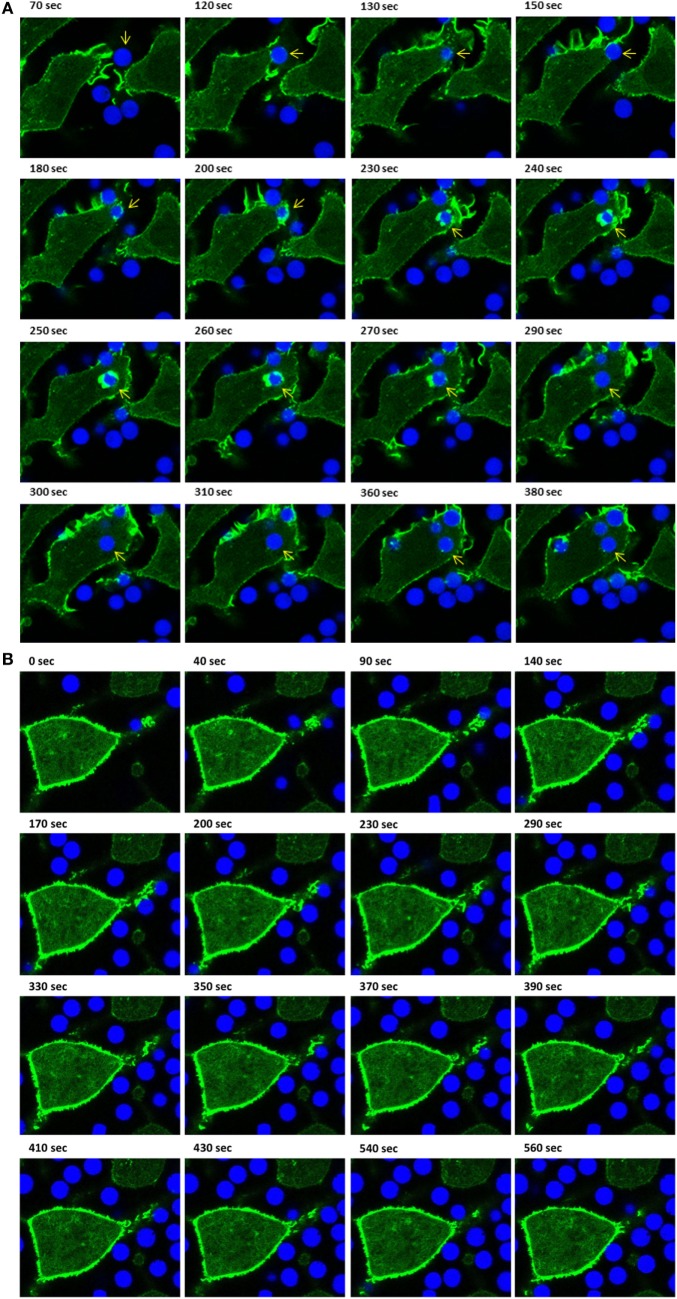
Live-cell imaging of IpLITR 2.6b/IpFcRγ-L-mediated phagocytosis at different incubation temperatures. Rat basophilic leukemia-2H3 cells (3 × 10^5^) stably co-expressing IpLITR 2.6b/IpFcRγ-L and LifeAct-GFP were incubated at 37°C **(A)** or at 27°C **(B)** with 9 × 10^5^ αHA monoclonal antibody-coated 4.5 µm bright blue microspheres. Immediately after the addition of target beads, images were collected at 10 s intervals for ~8 min using a Zeiss LSM 710 laser scanning confocal microscope (objective 60×, 1.3 oil plan-Apochromat; Munich, Germany). Representative time-stamps in **(A)** were extracted from Video S3 in Presentation 2 of Supplementary Material and the time-stamps in **(B)** were from Video S4 in Presentation 2 of Supplementary Material. In **(A)**, the target microsphere of interest is indicated with an arrowhead.

Next, we compared the phagocytic activities of IpLITR 2.6b/IpFcRγ-L-expressing cells incubated at 37 vs. 27°C. For these experiments, αHA-opsonized 4.5 µm BB were used as targets to allow for simultaneous visualization of both the target beads (blue) and F-actin dynamics (green). As shown in Video S3 in Presentation 2 of Supplementary Material and the accompanying time-stamped still images (Figure 3A; arrowhead), a microsphere target (blue) contacts the cell membrane (between 70–120 s) and is then progressively engulfed through a series of F-actin-mediated plasma membrane dynamic events similar to the temporal events described above. From initial contact to internalization, the entire process is completed in ~400 s at 37°C (Figure 3A). Note: although multiple beads are present in the LCI videos, for clarity we selected target beads that could be resolved from their initial contacts with the cell through to engulfment. An additional example of the IpLITR 2.6b/IpFcRγ-L-mediated phagocytosis process at 37°C is also shown in Video S4 in Presentation 2 of Supplementary Material with the time-stamped images shown in Figure S3 in Presentation 1 of Supplementary Material. In comparison, when IpLITR 2.6b/IpFcRγ-L-expressing RBL-2H3 cells were incubated with αHA-opsonized 4.5 µm BB at the inhibitory temperature of 27°C, no apparent target contacts or F-actin-mediated membrane dynamic events were observed. This is shown in Video S5 in Presentation 2 of Supplementary Material and the associated time-stamped images in Figure 3B that spans 560 s starting from the time when the targets were first introduced to the cells. Although several beads were observed in the field of view, none of these targets establish contacts with the cell membrane over the duration of the video.

### IpLITR 1.1b-Mediated Phagocytosis

**Figure 4:**
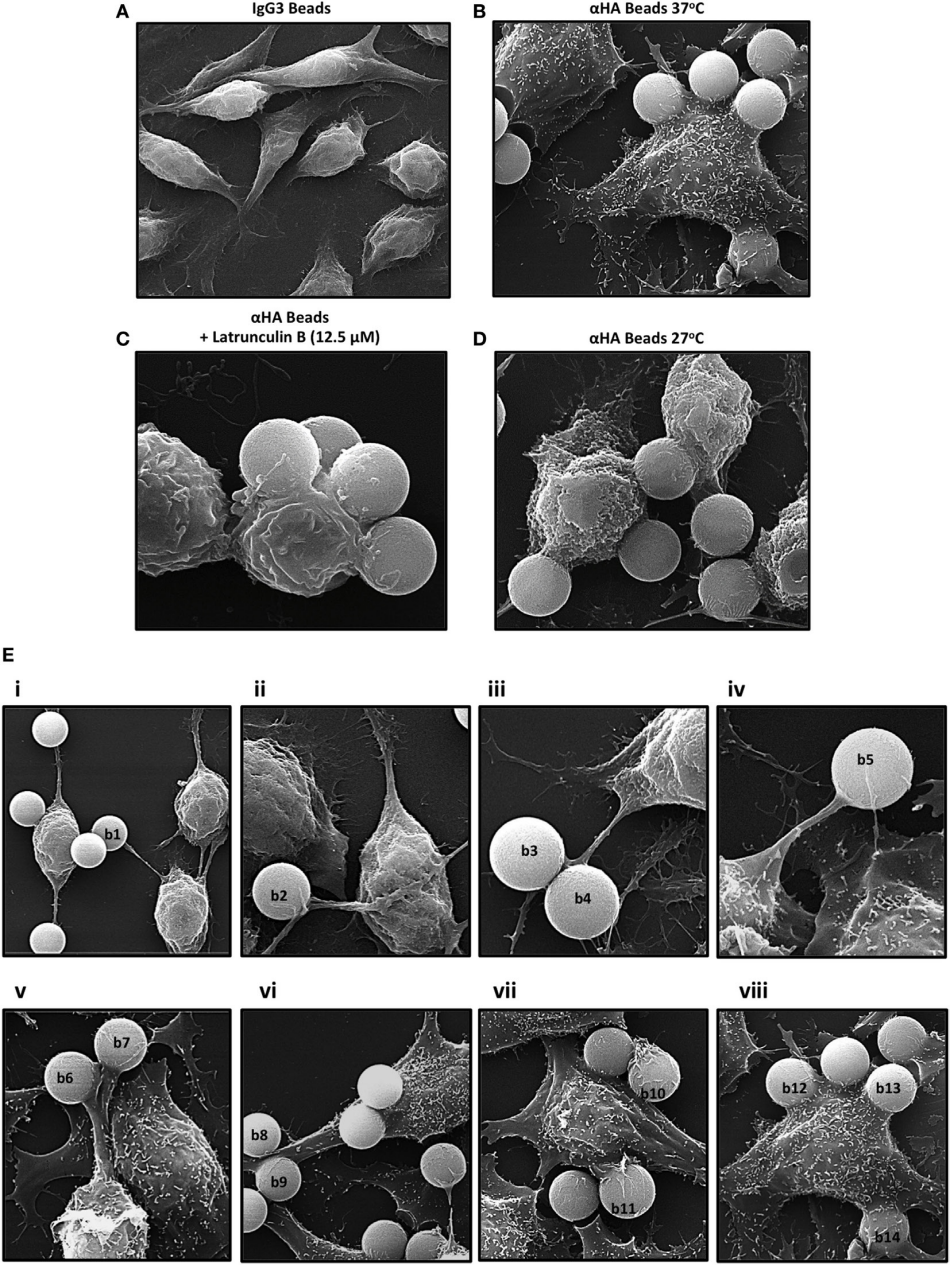
Scanning electron microscopy (SEM) of IpLITR 1.1b-mediated phagocytosis. IpLITR 1.1b-expressing rat basophilic leukemia-2H3 cells (3 × 10^5^) were incubated at 37°C for 1 h with 9 × 10^5^ IgG3-coated 4.5 µm microspheres **(A)** or with 9 × 10^5^ αHA monoclonal antibody (mAb)-coated 4.5 µm microspheres **(B)** prior to imaging using a Philips/FEI XL30 SEM microscope (FEI: Hillsboro, OR, USA). Cells (3 × 10^5^) were also pretreated for 1 h with 12.5 µM of the F-actin inhibitor Latrunculin B prior to their incubation with 9 × 10^5^ αHA mAb-coated 4.5 µm microspheres **(C)** or incubated at 27°C for 1 h with 9 × 10^5^ αHA mAb-coated 4.5 µm microspheres **(D)** prior to imaging. IpLITR 1.1b-expressing cells (3 × 10^5^) were also incubated at 37°C for various times (e.g., 4, 8, 16, and 32 min) with 9 × 10^5^ αHA mAb-coated 4.5 µm microspheres and representative SEM images of the progressive stages of IpLITR 1.1b-mediated phagocytosis are shown in panels i–viii **(E)**. Specific beads are labeled (b1–b14) as described in the results section.

Scanning electron microscopy was also performed to examine the IpLITR 1.1b-mediated phagocytic process (Figure 4). Similar to our observations for IpLITR 2.6b/IpFcRγ-L (Figure 1), when incubated with isotype control IgG3 beads, no internalized or surface-bound beads were observed (Figure 4A). However, when IpLITR 1.1b-expressing cells were incubated with αHA-opsonized beads (1 h at 37°C), SEM revealed that most of the targets appeared to be firmly secured to the cell surface but often they were not engulfed (Figure 4B). Pretreatment of the cells with the F-actin inhibitor Latrunculin B significantly altered cell morphology and also caused the beads to remain loosely tethered to the cell surface by disorganized plasma membrane structures (Figure 4C). When the IpLITR 1.1b-expressing cells were incubated with targets at 27°C for 1 h, the beads remained secured at the cell surface (Figure 4D), but the cells displayed a more rounded appearance compared to when they were incubated at 37°C. SEM imaging was also performed at 37°C for shorter time periods (i.e., 4, 8, 16, and 32 min) and Figure 4E shows representative images of the temporal stages of IpLITR 1.1b-mediated target interactions starting from initial cell-bead contacts (panels i–iv) through target capture and their eventual tethering to the cell membrane and occasional engulfment (panels v–viii). Specifically, during the early stages of target interactions most IpLITR 1.1b-expressing cells produced thin elongated membrane protrusions with beads tethered at their ends [Figure 4E; panels i-iv, beads (b1–b5)]. Some cells also generated thicker cellular extensions (Figure 4E; v, vi), which also participated in bead capture (b6–b9). We also consistently observed what appeared to be membrane ruffling (Figure 4E; vii), which contributed to the tethering of target beads to the plasma membrane (b10, b11). At later stages, stalled phagocytic cup-like structures (Figure 4E; viii) could be seen interacting with multiple beads (b12, b13) on the cell surface, and occasionally targets that were almost completely surrounded by the plasma membrane (b14). Please note that Figure 4E, panel viii, is the same image shown in Figure 4B.

### Temporal Examination of IpLITR 1.1b-Mediated Target Interactions

**Figure 5:**
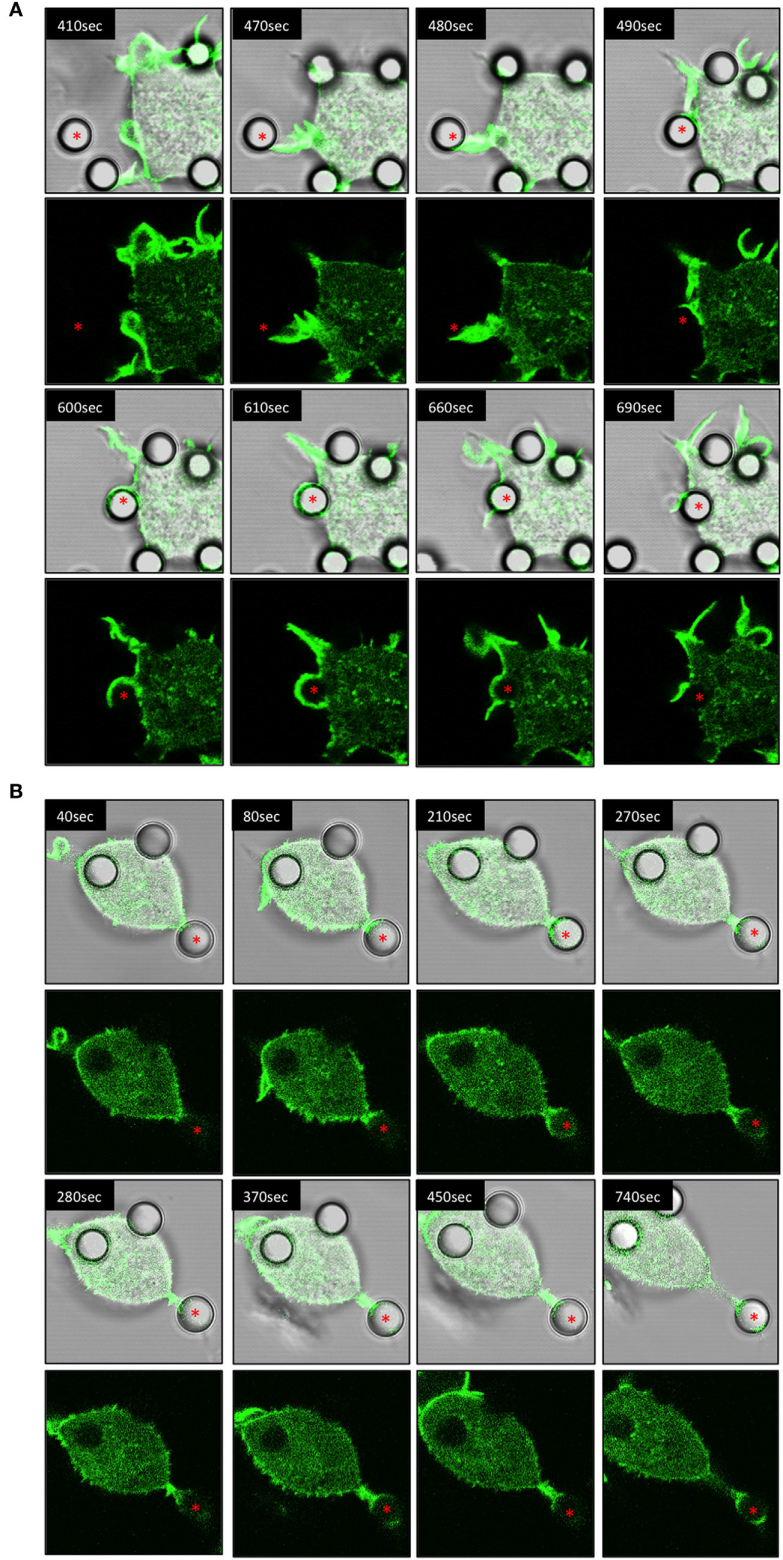
Live-cell imaging of IpLITR 1.1b-mediated target interactions. Rat basophilic leukemia-2H3 cells (3 × 10^5^) stably co-expressing IpLITR 1.1b and LifeAct-GFP were incubated at 37°C with 9 × 10^5^ αHA monoclonal antibody-coated 4.5 µm microspheres. Immediately after the addition of target beads, images were collected at 10 s intervals for ~8 min using a Zeiss LSM 710 laser scanning confocal microscope (objective 60×, 1.3 oil plan-Apochromat; Munich, Germany). Both the brightfield-LifeAct-GFP merged views (top panels) and the LifeAct-GFP views alone (bottom panels) are shown for two representative **(A,B)** IpLITR 1.1b-mediated target interactions with the location of the target microsphere indicated with an asterisk. Representative time-stamps in **(A,B)** were extracted from Videos S6 and S8 in Presentation 2 of Supplementary Material, respectively.

Using IpLITR 1.1b-expressing cells co-transfected with LifeAct-GFP, a variety of unique F-actin polymerization-dependent membrane remodeling events could be observed. Specifically, LCI imaging shows that IpLITR 1.1b-expressing cells generated F-actin-rich filopodia-like structures that extend out from the cell surface, attached to beads, and then rapidly withdrew back toward the cell membrane. For example, as shown in Video S6 in Presentation 2 of Supplementary Material and its associated time-stamped static images (Figure 5A; target marked with an asterisk), a representative IpLITR 1.1b-expressing cell produces a thick actin-rich extension (green) that reaches out and attaches to the target bead (Figure 5A; 410–480 s). After ~10 s of contact with the target, the bead is then rapidly retracted back toward the cell surface, which correlates with the disappearance of the F-actin-rich extension as shown in the time-stamped panels at 480–490 s (Figure 5A). Following ~100 s of sustained contact between the target bead and the plasma membrane, a second F-actin-rich pseudopod-like extension (Figure 5A; 600 s) can be seen crawling up and then over the outer edge of bead until it returns toward the cell surface; momentarily wrapping the target in the plasma membrane (Figure 5A; 600–610 s). Subsequently, the pseudopod then retracts away from the bead before rapidly disappearing with the bead now tethered at the cell surface (Figure 5A; 660–690 s). An alternative mode of filopodia-mediated capture of targets displayed by IpLITR 1.1b-expressing RBL-2H3 cells is also shown in Video S7 in Presentation 2 of Supplementary Material and its associated time-stamped images (Figure S4 in Presentation 1 of Supplementary Material). Here, the formation of a thin F-actin containing membrane protrusion (green) rapidly extends out from the cell surface and makes contact with a bead (Figure S4 in Presentation 1 of Supplementary Material; 60–110 s). After initial contact with the target, the membrane protrusion rapidly retracts back toward the cell surface (110–140 s), thus pulling the bead toward the cell and tethering it to the membrane. This is then followed by the transient generation of actin-dense pseudopod-like structures that appear to surround the bead (Figure S4 in Presentation 1 of Supplementary Material; 150–170 s). Another representative cell–target interaction phenotype that we only observed for IpLITR 1.1b-expressing cells using LCI was the generation of an F-actin-rich extended membranous stalk (Video S8 in Presentation 2 of Supplementary Material) that formed after initial contact with the bead (see Figure 5B; target bead indicated with an asterisk; at 40 s). Following target contact, this extended stalk exhibited probing behavior for ~700 s during which time there were variable levels of F-actin polymerization observed along the edges and around the surface of the bead (Figure 5B; 40–740 s). The plasma membrane stalk also appeared to both elongate and thicken over the course of its contact with the bead and, unlike what we described earlier (Figure 5A), the target remained at a distance from the cell body as it was not retracted back toward the membrane surface during the duration of the video (Video S8 in Presentation 2 of Supplementary Material). Of note, this IpLITR 1.1b-expressing cell also appeared to contain one internalized bead as well as one tethered bead in addition to the target bead we have documented in the time-lapse.

While IpLITR 1.1b-expressing cells commonly generated and used extended membranous F-actin containing structures to capture and tether extracellular targets, other dynamic interaction behaviors were also observed and representatives of these are shown in the Videos S9–S11 in Presentation 2 of Supplementary Material and their associated time-stamped images in Figures S5–S7 in Presentation 1 of Supplementary Material. For instance, IpLITR 1.1b-expressing cells were capable of generating complex membranous ruffles (Video S9 in Presentation 2 of Supplementary Material). In this example two extracellular beads are captured by actin-dense membrane ruffles generated at the cell surface (Figure S5 in Presentation 1 of Supplementary Material; red and yellow asterisks 130–270 s). After being captured, the formation of a second F-actin-rich membrane ruffle is observed, which encapsulates one of the beads (Figure S5 in Presentation 1 of Supplementary Material; yellow asterisk; 290–320 s) along the outer edge of the cell before depolymerizing as the bead is tethered to the cell surface (Figure S5 in Presentation 1 of Supplementary Material; 340 s). We also observed situations where a thin F-actin-dense protrusion captures a target bead at its outer most end (Video S10 in Presentation 2 of Supplementary Material). As the time-lapse progresses, polymerized actin accumulates around the bead as it is contracted down onto the cell surface (Figure S6 in Presentation 1 of Supplementary Material; 0–60 s). After this initial tethering, actin-dense membrane structures begin to surround the bead (Figure S6 in Presentation 1 of Supplementary Material; 110 s) initially from the left side and then from the right side of the target (Figure S6 in Presentation 1 of Supplementary Material; 170–200 s). Over the next 300 s, the F-actin depolymerizes leading to the apparent internalization of the bead (Figure S6 in Presentation 1 of Supplementary Material; 310–500 s). Finally, as shown in Video S11 in Presentation 2 of Supplementary Material; Figure S7 in Presentation 1 of Supplementary Material, multiple cell–bead interactions could be seen for an individual IpLITR 1.1b-expressing cell (Figure S7 in Presentation 1 of Supplementary Material; red, yellow, and orange asterisk). As the cell moves within the field of view from the top left, one bead had already begun to be internalized (Figure S7 in Presentation 1 of Supplementary Material; red asterisks; 160 s) and another bead was actively tethered to the cell surface (Figure S7 in Presentation 1 of Supplementary Material; yellow asterisks; 200–260 s). The generation of an F-actin-rich membrane protrusion then actively extended toward and contacted a third bead (Figure S7 in Presentation 1 of Supplementary Material; orange asterisks; 290–320 s). After this initial contact, a phagocytic-cup-like structure rapidly formed around the edges of the bead (Figure S7 in Presentation 1 of Supplementary Material; orange asterisks; 370 s), which was subsequently retracted back toward the cell surface (Figure S7 in Presentation 1 of Supplementary Material; 470 s). Overall, these results provide a representative summary of the diverse F-actin mediated plasma membrane remodeling events uniquely observed for IpLITR 1.1b- but not IpLITR 2.6b/IpFcRγ-L-expressing cells. Notably, most IpLITR 1.1b-mediated target interactions involved the formation of membranous extensions as further described below.

### IpLITR 1.1b-Mediated Target Interactions at 27°C

**Figure 6:**
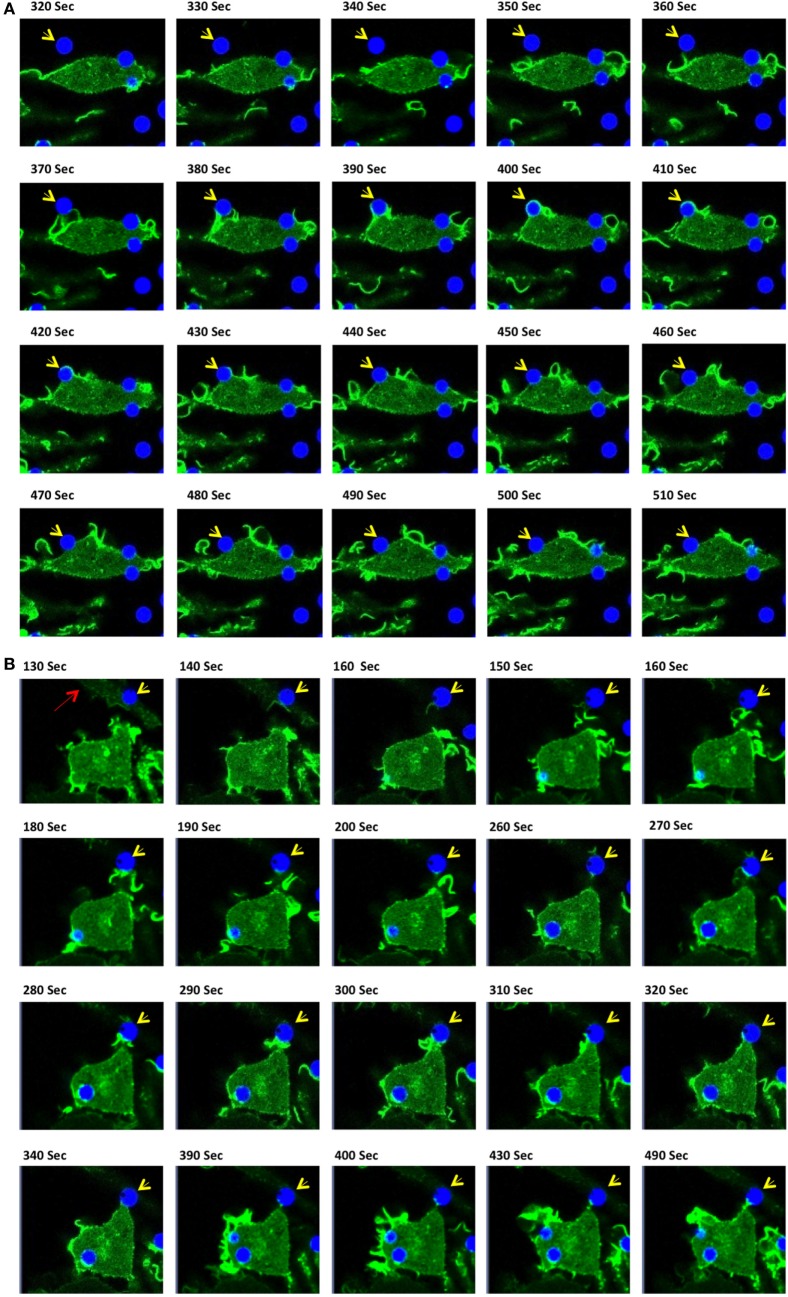

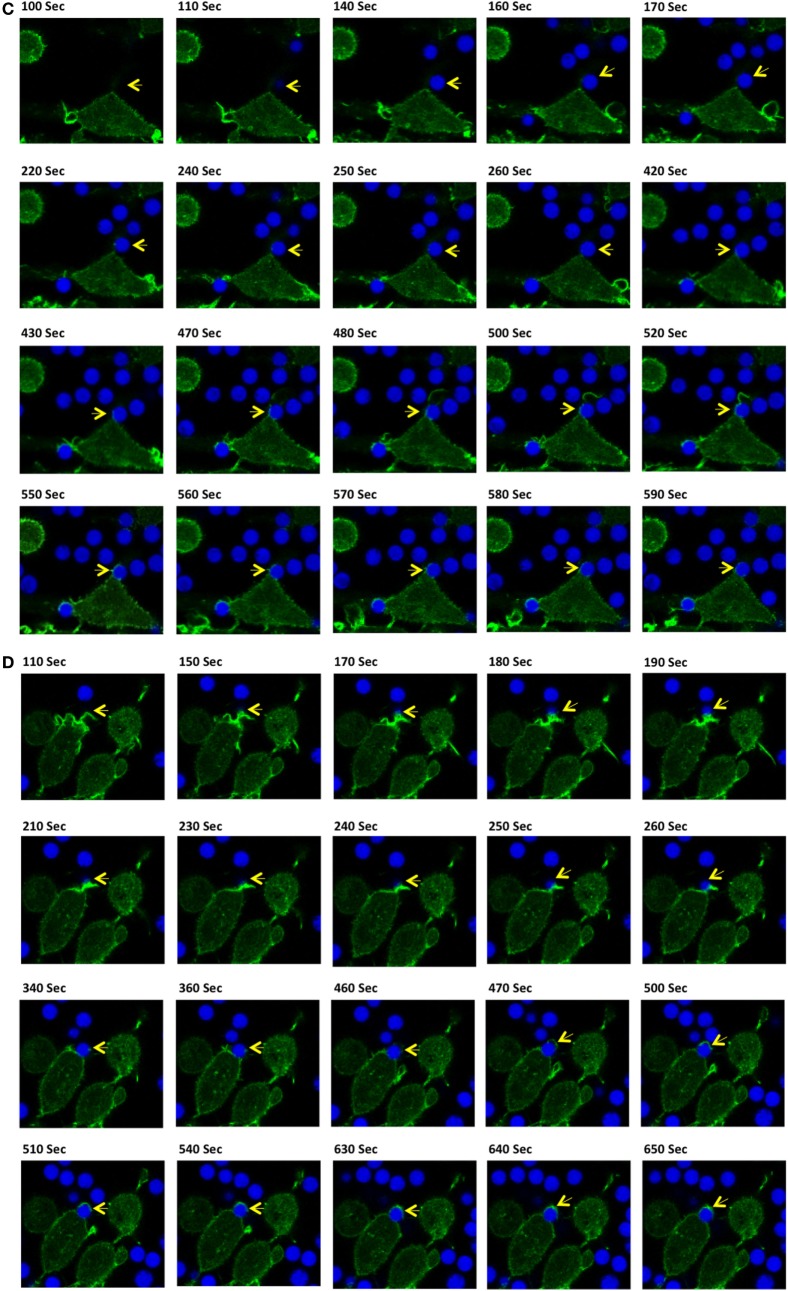

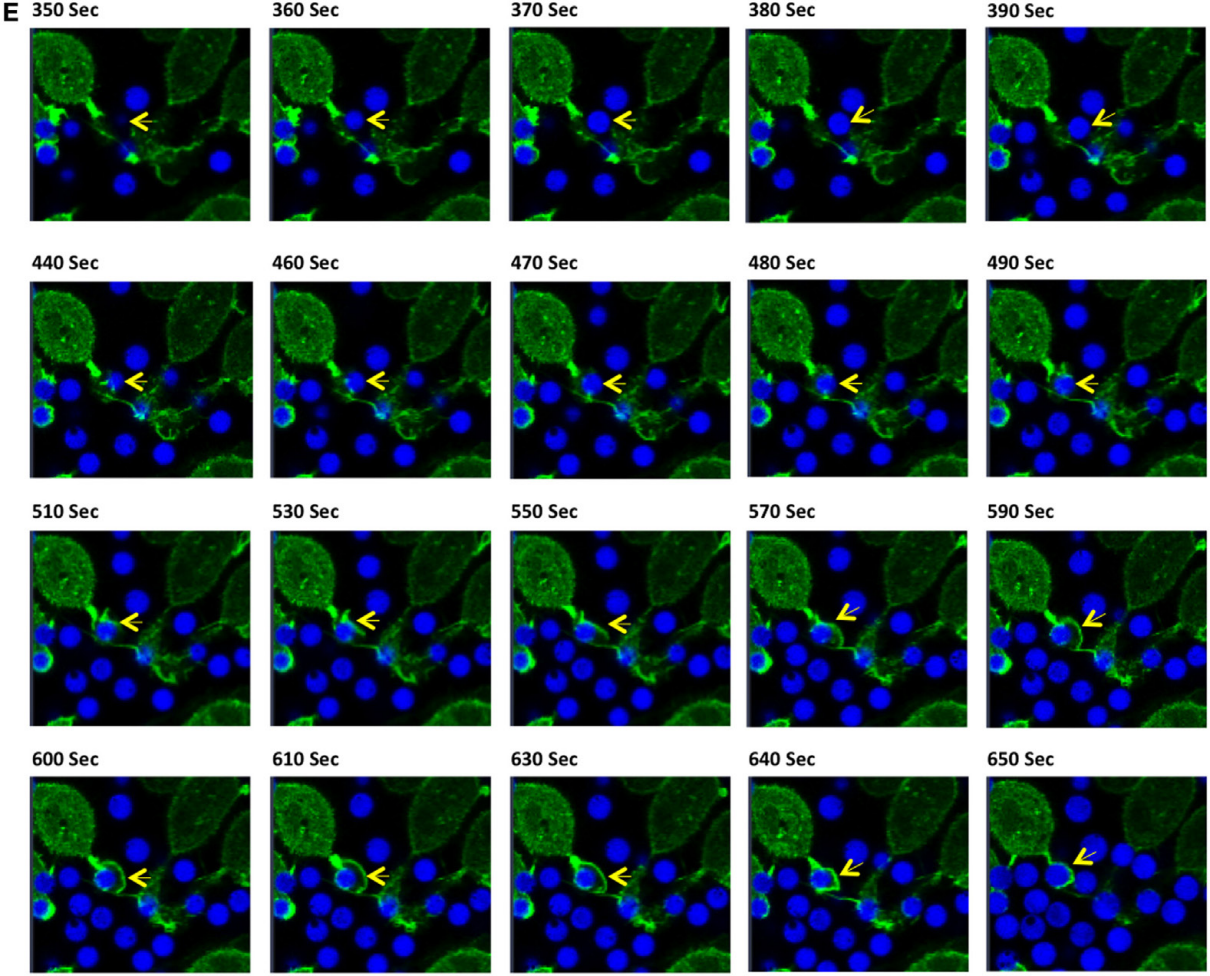
Live-cell imaging of IpLITR 1.1b-mediated target interactions at different incubation temperatures. Rat basophilic leukemia-2H3 cells (3 × 10^5^) stably co-expressing IpLITR 1.1b and LifeAct-GFP were incubated at 37°C **(A,B)** or at 27°C **(C–E)** with 9 × 10^5^ αHA monoclonal antibody-coated 4.5 µm bright blue microspheres. Immediately after the addition of target beads, images were collected at 10 s intervals for ~8 min using a Zeiss LSM 710 laser scanning confocal microscope (objective 60×, 1.3 oil plan-Apochromat; Munich, Germany). Representative time-stamps in **(A,B)** were extracted from Videos S12 in Presentation 2 and S15 in Presentation 3 of Supplementary Material, respectively, and the time-stamps in **(C–E)** were from Videos S16–S18 in Presentation 3 of Supplementary Material, respectively. In all time-stamps, target beads are indicated with an arrowhead and in (b; 130 s) a second cell with a pre-captured target bead is indicated with an arrow.

After documenting several unique phagocytic phenotypes for IpLITR 1.1b-expressing RBL-2H3 cells, experiments were then performed to compare IpLITR 1.1b-mediated target interactions at 37 vs. 27°C (Figure 6). The rationale for these experiments was based on our previously reported ability of IpLITR 1.1b-expressing cells to facilitate target interactions at lower incubation temperatures (29). Here, again, αHA-opsonized 4.5 µm BB were used to allow for simultaneous visualization of target beads (blue) and F-actin dynamics (green). As shown in an LCI time-lapse video (Video S12 in Presentation 2 of Supplementary Material) and the accompanying still images (Figure 6A; arrowhead), at 37°C an extracellular bead is located at a distance from the cell membrane until a series of F-actin-rich membrane structures (green) extend toward (Figure 6A; 350–360 s) and then contacts the target (Figure 6A; 370–380 s). The bead is then rapidly pulled toward the cell membrane during which time distinct actin polymerization events appear to mediate extension of pseudopods around the entire bead (Figure 6A; 390–400 s). The actin-rich pseudopod immediately retracts away from the bead as evidenced by the gradual depolymerization of F-actin from around the outer surface of the bead (Figure 6A; 410–440 s). Over the remainder of the time series, the captured bead remains tethered on the cell surface but it is not engulfed (Figure 6A; 450–510 s). There are two other targets in the frame that are similarly captured by this IpLITR 1.1b-expressing RBL-2H3 cell at 37°C, and their interactions are documented separately in Videos S13 and S14 in Presentation 3 of Supplementary Material and their accompanying time-stamped images (Figures S8 and S9 in Presentation 1 of Supplementary Material, respectively). In an additional representative cell–target interaction phenotype, we observed an IpLITR 1.1b-expressing cells that continuously probed and then repeatedly attempted to pull a pre-tethered microspheres away from another cell. Specifically, as shown in Video S15 in Presentation 3 of Supplementary Material, a target bead has already been captured and is visible at the top left of the video. As the time series progresses, a second cell visible in the center of the video projects an F-actin containing membrane ruffle toward this target (Figure 6B, arrowhead; 130–180 s), which is already tethered to the other cell (Figure 6B, arrow; 130 s). After the ruffle makes initial contact with this bead, it appears to then withdraw back toward the cell leaving an extended membrane structure attached to the target with a detectable F-actin-rich area at the point of contact with the bead (Figure 6B; 190–260 s). Subsequently, following the cells initial failed attempt to pull the bead back toward the cell, a secondary F-actin-rich membrane ruffle projects out toward the secured target (Figure 6B; 270–300 s). As this ruffle subsides and the F-actin depolymerizes, the cell has again failed to retract the target toward its surface, although contact with this bead still remains (Figure 6B; 310–490 s). When viewed in its entirety, three separate F-actin-rich membrane ruffles are actively projected toward the target in what may be repeated attempts to capture the tethered target. Ultimately, these events leave the bead attached to two separate cells. Of note, this cell also appears to have engulfed two other targets as viewed in the bottom left of the video (Figure 6B; 430 s).

Unlike the inhibition of phagocytic responses we observed for IpLITR 2.6b/IpFcRγ-L at lower incubation temperatures, IpLITR 1.1b-expressing cells continued to display active target capture phenotypes at 27°C. However, at this lower incubation temperature the overall activity of the F-actin mediated membrane dynamics were markedly repressed. For example, as shown Video S16 in Presentation 3 of Supplementary Material and associated time-stamped images (Figure 6C), an extracellular target (arrowhead) is initially contacted by the cell (Figure 6C; 430 s), which promotes the extension of plasma membrane around the left side of the target (Figure 6C; 430–470 s). This appears to promote the formation of a thin F-actin-rich structure that extends beyond the surface of the attached bead and into the extracellular space (Figure 6C; 480–500 s). At this stage, the membrane protrusion appears to momentarily probe the environment before collapsing back toward the cell, which coincides with the disappearance of the F-actin signal (Figure 6C; 520–550 s). At the conclusion of this time series, the bead remains tethered at the cell surface but it was not engulfed (Figure 6C; 590 s). Shown in Video S17 in Presentation 3 of Supplementary Material and Figure 6D (time-stamped images) is another example at 27°C where IpLITR 1.1b-expressing cells first use an F-actin containing membrane ruffle to attach to a bead, which is then followed by the formation of a thin F-actin containing membrane structure that partially surrounds the tethered target. In Video S18 in Presentation 3 of Supplementary Material and Figure 6E (time-stamped images) a target bead is observed coming into close contact with an extended membrane protrusion, after which it is then partially engulfed as it becomes surrounded by a thin but F-actin-dense membrane structure. Additional examples of the unique activities of IpLITR 1.1b-expressing RBL-2H3 cells at 27°C are provided in Videos S19 and S20 in Presentation 3 of Supplementary Material and their accompanying time-stamped images (Figures S10 and S11 in Presentation 1 of Supplementary Material, respectively).

## Discussion

Using high-resolution SEM and real-time LCI, the results of this study provide new evidence regarding IpLITR-mediated production of dynamic cytoskeletal and membrane remodeling events. In particular, we show for the first time that IpLITR 1.1b-expressing cells uniquely generate filopodia-like extensions composed of long filaments of polymerized F-actin and reveal that these plasma membrane structures are actively used for extracellular target binding and capture. Considering that very little is known regarding the ability of immunoregulatory receptor-types to induce filipodia formation in other vertebrates, including mammals, our functional studies described here set the stage for future studies targeted at understanding how the dynamic control of intracellular transduction events controlled by IpLITR 1.1b selectively contribute to diverse innate cell effector responses across vertebrates. That being said, it is important to note that our results were obtained using heterologous expression of fish immunoregulatory proteins in a mammalian cell system. Although this strategy is not directly able to inform us about the actual *in vivo* activities of IpLITRs, it is clear from our studies that these receptors can potently regulate various innate cellular responses including the selective induction of filopodia for extracellular target capture. It is likely that IpLITR-mediated responses in mammalian cells feature similar signaling components that would be present in representative fish immune cell-types. Further exploration of these mechanisms will assist in uncovering the functional versatility for ITIM- and ITAM-encoding receptors that can eventually be used to explore teleost immunoregulatory receptor networks in homologous systems.

Filopodia are dynamic membrane structures that can vary in length and thickness but rely on the cytoskeletal machinery and actin-binding proteins for their formation (10–13, 19). Filopodia also play many important physiological roles in health and disease and have been shown to participate in a range of cellular processes including cell migration, morphogenesis, neurite outgrowth, metastasis, and wound healing (10–13, 19, 35–37). For example, within the immune system, SEM analysis has shown that prior to the initiation of phagocytosis, bacteria are tethered to phagocyte surfaces by long and thin membranous protrusions (11–14, 17, 18). Importantly, these plasma membrane extensions provide phagocytes with the ability to dynamically explore their extracellular environments as the rapid elongation and subsequent retraction of filopodia assists in the active capture of microbes by increasing the functional radius available for pathogen contact beyond the circumference of the cell (1, 7, 11–14, 18). While this shows that immune cells can actively deploy filopodia to capture targets, very little is known regarding the specific receptor-types and associated intracellular dynamics that participate in the formation and regulation of these membrane structures. Previously, we have reported that IpLITR 1.1b-expressing RBL-2H3 cells displayed a unique target acquisition and engulfment phenotype associated with the formation of extended membranous protrusions (29). The results of the present study further support a role for this specific immunoregulatory receptor-type in the control of cytoskeletal dynamics and filopodia formations during the initial contact with and then capture of extracellular targets over a range of temperatures. Taken together, these findings show, for the first time, that active capture and tethering of extracellular targets to the cell surface might represent a conserved function for certain members of the IpLITR family *via* their unique ability to transmit signals that affect F-actin polymerization and associated plasma membrane dynamics. Comparatively, for IpLITR 2.6b/IpFcRγ-L-expressing cells, filopodia-like structures were not specifically used to capture targets, as sustained contact time between the plasma membrane and microspheres was required to trigger the IpLITR 2.6b/IpFcRγ-L-mediated phagocytic process. In addition, phagocytic activity and membrane dynamics were both completely abolished at 27°C in IpLITR 2.6b/IpFcRγ-L-expressing cells; likely due to an inability of IpLITR 2.6b/IpFcRγ-L to promote or facilitate F-actin polymerization events at temperatures below 37°C in RBL-2H3 cells.

Filopodia are regulated by mechanisms instigated in part by constitutive intracellular signaling events that involve a number of conserved transduction molecules (e.g., docking protein 1, non-catalytic region of tyrosine kinase adaptor protein 1 (Nck), neural Wisskot–Aldrich syndrome protein, N-WASp family verprolin-homologous protein-2 (Wave2), inverse-BAR protein insulin receptor substrate protein of 53 kDa, Cdc42, formins, fascin, and myosins) (10, 19, 38–44). Overall, constitutively generated filopodia allow phagocytes to constantly probe their extracellular environments as these membranous probes also contain phagocytic receptors located along their edges, a process that depends on an unknown mechanism for the loading of phagocytic receptors into the protrusions (1, 18, 22). Any stochastic phagocytic receptor–target interactions that may occur would facilitate the attachment of specific targets to the extended membranes. Extracellular targets would then be pulled back toward the cell surface during filopodial retractions due to the retrograde flow of actin back toward the cell body and the contractile forces generated by myosins (10). Once in close contact with the plasma membrane, newly established target–receptor interactions could activate additional intracellular signaling pathways to reinforce tethering or subsequently trigger target engulfment (7, 18).

While constitutively generated filopodia facilitates continuous sampling of the environment by phagocytes, it has been shown that these structures are also produced in responses to specific stimuli. For example, lipopolysaccharide-induced activation of toll-like receptor 4 increases the production of filopodia-like structures (15, 16). Receptor-induced filopodia formation has also been characterized in cancer cells, which use these inducible pathways to produce invasive membrane protrusions (36, 37). Termed invadopodia, these extensions are formed by the selective stimulations of tumor cell-expressed platelet-derived growth factor and epidermal growth factor (36). Upon growth factor stimulation, the local recruitment and activation of kinases, such as focal adhesion kinase and proto-oncogene tyrosine-protein kinase Src (Src), occurs early in the process of invadopodia formation that initiate phosphorylation of downstream signaling proteins (36, 37). Unlike the receptor-specific production of filopodia or invadopodia described above, IpLITR 1.1b-expressing cells were not stimulated by any known endogenous ligands. Therefore, it seems that the stable expression of IpLITR 1.1b alone was sufficient to support filopodia generation. These results suggest that IpLITR 1.1b can uniquely network with intracellular components requisite for the production of F-actin containing filopodia-like structures. Previously, we hypothesized that IpLITR 1.1b-controlled signaling events induce formation of macromolecular complexes with its CYT that pre-assemble prior to receptor engagement; effectively priming the receptor for subsequent interactions with extracellular targets (28–30). Pre-associations of IpLITR 1.1b with intracellular effectors capable of modulating the cytoskeletal machinery would allow for dynamic membrane remodeling events prior to the formation of stable receptor–ligand interactions (28–30).

Our proposed mechanism for target acquisition and engulfment pathways facilitated by IpLITR 1.1b have been described in detail elsewhere (28, 29, 45) and we hypothesized that this likely requires the differential participation of the proximal and distal regions of its CYT in the recruitment and activation of select intracellular effectors (45, 46). Specifically, for IpLITR 1.1b to constitutively trigger filopodia formation in RBL-2H3 cells without ligand engagements, this receptor may exist in a primed state, facilitating its basal coupling to effectors of actin dynamics. In this model, Nck serves as a cytosolic adaptor that could couple surface expressed IpLITR 1.1b with the intracellular effector Wave2. Basal recruitment and activation of a cytoplasmic guanine nucleotide exchange factor such a proto-oncogene Vav (Vav) family proteins would then activate Rho family GTPases (47), which may then activate F-actin polymerization *via* the Nck-associated Wave2 complex to trigger the constitutive formation of filopodia. Interestingly, this model closely aligns with the short-circuited phagocytic pathway recently described for human carcinoembryonic antigen-related cell adhesion molecule (48). In addition, we have reported that IpLITR 1.1b-mediated activity is partially dependent upon the catalytic activity of Src and the spleen tyrosine kinase (Syk) (29). Although yet to be confirmed, sustained activation of these kinases in the absence of agonist stimulation would likely require pre-aggregation of IpLITR 1.1b on the cell surface. This would maintain basal Src-dependent tyrosine phosphorylation of the IpLITR 1.1b CYT region facilitating constitutive coupling of IpLITR 1.1b to select components of the cytoskeletal machinery. Importantly, we also recently showed that Nck is recruited to a consensus interaction motif located in the proximal CYT region of IpLITR 1.1b (45), which would directly bridge IpLITR 1.1b with the Wave2 complex. In mammalian cells, activation of Wave2 requires state-specific phosphorylation as well as interactions with GTP-bound Rho superfamily proteins, most commonly Rac (49). As a result, the assembly of the Nck-Wave2 complex within the proximal CYT region of IpLITR 1.1b would most likely be coupled to the recruitment of cytoplasmic guanine nucleotide exchange factors, including Vav. Our recent biochemical studies showed that Syk is preferentially recruited to the distal region of the IpLITR 1.1b CYT (45). Therefore, we suspect that recruitment and activation of Vav by Syk would provide the necessary catalyst for Rac 1/2 activation and the stimulation of actin-driven membrane protrusions *via* the Nck-recruited Wave2. Overall, this predicted model encompasses the minimal machinery required for a constitutive IpLITR 1.1b-dependent deployment of filopodia in the absence of agonist stimulation and is supported by our recent biochemical studies (27, 29, 45). Future work is required to formally establish functional roles for Nck, Syk, Vav, Rac 1/2, and Wave2 during IpLITR 1.1b-mediated triggering responses including filopodia formation. Finally, if IpLITR 1.1b is indeed basally phosphorylated and pre-associated with intracellular components linking it to F-actin dynamics, then this would in part explain why IpLITR 1.1b continues to capture targets at suboptimal incubation temperatures due to pre-assembly of these components with the receptor. The reduced plasma membrane dynamics for IpLITR 1.1b-expressing RBL-2H3 cells at 27°C are likely due to specific affects on phospholipid dynamics and membrane mobility at this lower temperature, but likely not from an inability of IpLITR 1.1b to associate with signaling complexes, which would have previously occurred prior to the cooling of the cells.

Following filopodia-mediated capture of extracellular targets, we frequently observed the generation of secondary waves of F-actin polymerization after the target was secured at the cell surface. These events may be triggered by aggregations of IpLITR 1.1b at the newly established contact sites formed between the plasma membrane and the captured target. In some cases, the immobilized targets remained firmly tethered on the cell surface and occasionally the beads were completely internalized. This phenotype is reminiscent of efferocytosis, a process responsible for phagocyte-mediated clearance of apoptotic bodies through the recognition of phosphatidylserine (PtdSer) on dying cells (50, 51). However, unlike linear filopodia that extend perpendicular to the cell surface, efferocytosis typically involves membrane dynamics that form extended but laterally moving arcs or wave-like structures that flow along the cell surface (50–52). Functionally, these structures reach out into the extracellular space to make contact with apoptotic cells and their sweeping motion augments trapping of distant targets. This brings dying cells into close proximity to the plasma membrane, where they are tethered and eventually cleared by secondary activated phagocytic processes (50, 51). Rac 1/2, Cdc42, and Wave2 have all been identified as key players during the control of efferocytosis (50, 51, 53, 54), which occurs in two discrete receptor-specific steps known as the tethering and tickling (50, 53, 55, 56) that participate in the step-wise capture and engulfment of apoptotic bodies. For example, engagement of receptors for identifying apoptotic cells, including CD36, CD14, CD68, ανβ3, and ανβ5, promotes the tethering of specific targets on macrophages (56). Uptake of targets then occurs when tethering receptors are co-engaged with the phagocytic PtdSer receptor (PSR) (56). Interestingly, incubation of the cells with PtdSer-coated erythrocytes was insufficient for both tethering and phagocytic uptake by the PSR; indicating that both tethering and phagocytic signals are required for effective apoptotic cell removal (56). In agreement with this dual mode for target capture and engulfment, our observations support a model that involves constitutive mechanisms for IpLITR 1.1b-mediated deployment of filopodia to tether targets to the cell surface. Subsequently, captured targets can trigger additional IpLITR 1.1b-dependent pathways, which may be distinct from the constitutive mechanism that regulates resting filopodia production. One example includes a CYT proximal-specific pathway involving the formation of a heterotrimeric complex consisting of growth factor receptor-bound protein, growth factor receptor-bound protein-associated binding protein 2 and phosphoinositide 3-kinases (57–60) that recruits Vav to activate Rac 1/2 and then trigger the actin-related protein 2/3-dependent actin protrusions *via* the Wave complex. Unlike constitutive filopodia induction, this model would be distinct from basal Nck-mediated recruitment of the actin regulatory Wave2 complex and could be achieved *via* the localized production of phosphatidylinositol 3,4,5-trisphosphate (61). A second example suggests that the phosphorylation of protein tyrosine phosphatases at the C-terminal tyrosine residue Y_542_ may form a cryptic ITAM in concert with a neighboring IpLITR 1.1b present at the site of bead contact to recruit phosphorylated protein tyrosine phosphatases. This mechanism would be similar to the recently discovered pathway described for dectin-1 (62) and would require only the distal segment of IpLITR 1.1b CYT. Future studies are still required to decipher the specific mechanisms underlying the variable signaling events that control IpLITR 1.1b-mediated regulation of target capture, tethering, and engulfment. However, the results of this study combined with our previous biochemical recruitment experiments provide the necessary framework for deciphering how IpLITR 1.1b variably controls the actin polymerization machinery.

Taken together, our results show that the expression of IpLITR 1.1b, but not IpLITR 2.6b/IpFcRγ-L, specifically triggers RBL-2H3 cells to induce filopodia formation in the absence of any known immune stimuli. The receptor-specific nature of IpLITR 1.1b-indcued filopodia is clearly evident when both IpLITR-expressing cell-types were incubated at depressed temperatures. This also appears to be the first study to suggest that expression of a specific immunoregulatory receptor can promote the constitutive formation of filopodia without the need for an exogenous ligand. IpLITR 1.1b-mediated signaling also initiates secondary waves of actin polymerization events that are associated with the internalization and membrane tethering of extracellular targets, which we propose to be a distinct event from those involved in the initial generation of filopodia. These responses are likely due to the unique structure and signaling potential associated with the IpLITR 1.1b CYT; thereby allowing for diversity in the integrated control of cytoskeletal and membrane remodeling associated with IpLITR 1.1b expression. Overall, our results offer novel information regarding the ability of immunoregulatory receptors to initiate filopodia formation and provide new insights into the temporal organization of cellular events surrounding the unique transduction dynamics that regulate F-actin polymerization and membrane remodeling events.

## Author Contributions

JS and DL conceived and designed the study. DL performed the experimental procedures. DL, JP, and JS analyzed the data, wrote the manuscript, and reviewed the manuscript.

## Conflict of Interest Statement

The authors declare that the research was conducted in the absence of any commercial or financial relationships that could be construed as a potential conflict of interest.

## Supplementary Material

Figure S1 Flow cytometric analysis of IpLITR surface expression levels in rat basophilic leukemia-2H3 (RBL-2H3) cells co-expressing LifeAct-GFP. IpLITR 2.6b/IpFcRγ-L **(A)** and IpLITR 1.1b **(B)**-expressing RBL-2H3 stables were transfected with LifeAct-GFP by nucleofection using the Amaxa Cell Line Nucleofector Kit T, RBL-2H3. IpLITR surface expression (FL-2) was determined by staining the cells with αHA monoclonal antibody (mAb) and a PE-conjugated secondary goat αmouse polyclonal. LifeAct-GFP expression was examined using the FL-1 intensity. Each histogram shows the staining profiles for cells stained with an IgG3 isotype control antibody (solid line), IpLITR-expressing cells stained with αHA mAb (untransfected; dotted line), and IpLITR-expressing cells co-transfected with LifeAct-GFP and stained with αHA mAb (LifeAct-GFP Transfected; dashed line).

Figure S2 Live-cell imaging of IpLITR 2.6b/IpFcRγ-L-mediated phagocytosis. Rat basophilic leukemia-2H3 cells (3 × 10^5^) stably co-expressing IpLITR 2.6b/IpFcRγ-L and LifeAct-GFP were incubated at 37°C with 9 × 10^5^ αHA monoclonal antibody-coated 4.5 µm microspheres. Immediately after the addition of target beads, images were collected at 10 s intervals for ~8 min using a Zeiss LSM 710 laser scanning confocal microscope (objective 60×, 1.3 oil plan-Apochromat; Munich, Germany). Both the brightfield-LifeAct-GFP merged views (top panels) and the LifeAct-GFP views alone (bottom panels) are shown with the location of the target microsphere indicated with an asterisk. Representative time-stamps were extracted from Video S2 in Supplementary Material.

Figure S3 Live-cell imaging of IpLITR 2.6b/IpFcRγ-L-mediated phagocytosis. Rat basophilic leukemia-2H3 cells (3 × 10^5^) stably co-expressing IpLITR 2.6b/IpFcRγ-L and LifeAct-GFP were incubated at 37°C with 9 × 10^5^ αHA monoclonal antibody-coated 4.5 µm bright blue microspheres. Immediately after the addition of target beads, images were collected at 10 s intervals for ~8 min using a Zeiss LSM 710 laser scanning confocal microscope (objective 60×, 1.3 oil plan-Apochromat; Munich, Germany). Representative time-stamps were extracted from Video S4 in Supplementary Material.

Figure S4 Live-cell imaging of IpLITR 1.1b-mediated target interactions. Rat basophilic leukemia-2H3 cells (3 × 10^5^) stably co-expressing IpLITR 1.1b and LifeAct-GFP were incubated at 37°C with 9 × 10^5^ αHA monoclonal antibody-coated 4.5 µm microspheres. Immediately after the addition of target beads, images were collected at 10 s intervals for ~8 min using a Zeiss LSM 710 laser scanning confocal microscope (objective 60×, 1.3 oil plan-Apochromat; Munich, Germany). Both the brightfield-LifeAct-GFP merged views (top panels) and the LifeAct-GFP views alone (bottom panels) are shown with the location of the target microsphere indicated with an asterisk. Representative time-stamps were extracted from Video S7 in Supplementary Material.

Figure S5 Live-cell imaging of IpLITR 1.1b-mediated target interactions. Rat basophilic leukemia-2H3 cells (3 × 10^5^) stably co-expressing IpLITR 1.1b and LifeAct-GFP were incubated at 37°C with 9 × 10^5^ αHA monoclonal antibody-coated 4.5 µm microspheres. Immediately after the addition of target beads, images were collected at 10 s intervals for ~8 min using a Zeiss LSM 710 laser scanning confocal microscope (objective 60×, 1.3 oil plan-Apochromat; Munich, Germany). Both the brightfield-LifeAct-GFP merged views (top panels) and the LifeAct-GFP views alone (bottom panels) are shown with the locations of two target microspheres indicated with asterisks. Representative time-stamps were extracted from Video S9 in Supplementary Material.

Figure S6 Live-cell imaging of IpLITR 1.1b-mediated target interactions. Rat basophilic leukemia-2H3 cells (3 × 10^5^) stably co-expressing IpLITR 1.1b and LifeAct-GFP were incubated at 37°C with 9 × 10^5^ αHA monoclonal antibody-coated 4.5 µm microspheres. Immediately after the addition of target beads, images were collected at 10 sec intervals for ~8 min using a Zeiss LSM 710 laser scanning confocal microscope (objective 60×, 1.3 oil plan-Apochromat; Munich, Germany). Both the brightfield-LifeAct-GFP merged views (top panels) and the LifeAct-GFP views alone (bottom panels) are shown with the location of the target microsphere indicated with an asterisk. Representative time-stamps were extracted from Video S10 in Supplementary Material.

Figure S7 Live-cell imaging of IpLITR 1.1b-mediated target interactions. Rat basophilic leukemia-2H3 cells (3 × 10^5^) stably co-expressing IpLITR 1.1b and LifeAct-GFP were incubated at 37°C with 9 × 10^5^ αHA monoclonal antibody-coated 4.5 µm microspheres. Immediately after the addition of target beads, images were collected at 10 s intervals for ~8 min using a Zeiss LSM 710 laser scanning confocal microscope (objective 60×, 1.3 oil plan-Apochromat; Munich, Germany). Both the brightfield-LifeAct-GFP merged views (top panels) and the LifeAct-GFP views alone (bottom panels) are shown with the locations of three target microspheres indicated with asterisks. Representative time-stamps were extracted from Video S11 in Supplementary Material.

Figure S8 Live-cell imaging of IpLITR 1.1b-mediated target interactions. Rat basophilic leukemia-2H3 cells (3 × 10^5^) stably co-expressing IpLITR 1.1b and LifeAct-GFP were incubated at 37°C with 9 × 10^5^ αHA monoclonal antibody-coated 4.5 µm bright blue microspheres. Immediately after the addition of target beads, images were collected at 10 s intervals for ~8 min using a Zeiss LSM 710 laser scanning confocal microscope (objective 60×, 1.3 oil plan-Apochromat; Munich, Germany). Target bead of interest is indicated with an arrowhead. Representative time-stamps were extracted from Videos S13 in Supplementary Material.

Figure S9 Live-cell imaging of IpLITR 1.1b-mediated target interactions. Rat basophilic leukemia-2H3 cells (3 × 10^5^) stably co-expressing IpLITR 1.1b and LifeAct-GFP were incubated at 37°C with 9 × 10^5^ αHA monoclonal antibody-coated 4.5 µm bright blue microspheres. Immediately after the addition of target beads, images were collected at 10 s intervals for ~8 min using a Zeiss LSM 710 laser scanning confocal microscope (objective 60×, 1.3 oil plan-Apochromat; Munich, Germany). Target bead of interest is indicated with an arrowhead. Representative time-stamps were extracted from Videos S14 in Supplementary Material.

Figure S10 Live-cell imaging of IpLITR 1.1b-mediated target interactions at 27°C. Rat basophilic leukemia-2H3 cells (3 × 10^5^) stably co-expressing IpLITR 1.1b and LifeAct-GFP were incubated at 27°C with 9 × 10^5^ αHA monoclonal antibody-coated 4.5 µm bright blue microspheres. Immediately after the addition of target beads, images were collected at 10 s intervals for ~8 min using a Zeiss LSM 710 laser scanning confocal microscope (objective 60×, 1.3 oil plan-Apochromat; Munich, Germany). Target bead of interest is indicated with an arrowhead. Representative time-stamps were extracted from Videos S19 in Supplementary Material.

Figure S11 Live-cell imaging of IpLITR 1.1b-mediated target interactions at 27°C. Rat basophilic leukemia-2H3 cells (3 × 10^5^) stably co-expressing IpLITR 1.1b and LifeAct-GFP were incubated at 27°C with 9 × 10^5^ αHA monoclonal antibody-coated 4.5 µm bright blue microspheres. Immediately after the addition of target beads, images were collected at 10 s intervals for ~8 min using a Zeiss LSM 710 laser scanning confocal microscope (objective 60×, 1.3 oil plan-Apochromat; Munich, Germany). Target bead of interest is indicated with an arrowhead. Representative time-stamps were extracted from Videos S20 in Supplementary Material.

Video S1 Live-cell imaging of IpLITR 2.6b/IpFcRγ-L-mediated phagocytosis. Rat basophilic leukemia-2H3 cells stably co-expressing IpLITR 2.6b/IpFcRγ-L and LifeAct-GFP were incubated at 37°C with αHA monoclonal antibody-coated 4.5 µm microspheres. Immediately after the addition of target beads, images were collected at 10 s intervals for ~8 min using a Zeiss LSM 710 laser scanning confocal microscope. Both the brightfield-LifeAct-GFP merged views **(A)** and the LifeAct-GFP views alone **(B)** are shown. Target bead of interest is located within the box.

Video S2 Live-cell imaging of IpLITR 2.6b/IpFcRγ-L-mediated phagocytosis. Rat basophilic leukemia-2H3 cells stably co-expressing IpLITR 2.6b/IpFcRγ-L and LifeAct-GFP were incubated at 37°C with αHA monoclonal antibody-coated 4.5 µm microspheres. Immediately after the addition of target beads, images were collected at 10 s intervals for ~8 min using a Zeiss LSM 710 laser scanning confocal microscope. Both the brightfield-LifeAct-GFP merged views **(A)** and the LifeAct-GFP views alone **(B)** are shown. Target bead of interest is located within the box.

Video S3 Live-cell imaging of IpLITR 2.6b/IpFcRγ-L-mediated phagocytosis. Rat basophilic leukemia-2H3 cells stably co-expressing IpLITR 2.6b/IpFcRγ-L and LifeAct-GFP were incubated at 37°C with αHA monoclonal antibody-coated 4.5 µm bright blue microspheres. Immediately after the addition of target beads, images were collected at 10 s intervals for ~8 min using a Zeiss LSM 710 laser scanning confocal microscope. Target bead of interest is located within the box.

Video S4 Live-cell imaging of IpLITR 2.6b/IpFcRγ-L-mediated phagocytosis. Rat basophilic leukemia-2H3 cells stably co-expressing IpLITR 2.6b/IpFcRγ-L and LifeAct-GFP were incubated at 37°C with αHA monoclonal antibody-coated 4.5 µm bright blue microspheres. Immediately after the addition of target beads, images were collected at 10 s intervals for ~8 min using a Zeiss LSM 710 laser scanning confocal microscope. Target bead of interest is located within the box.

Video S5 Live-cell imaging of IpLITR 2.6b/IpFcRγ-L-mediated phagocytosis at 27^o^C. Rat basophilic leukemia-2H3 cells stably co-expressing IpLITR 2.6b/IpFcRγ-L and LifeAct-GFP were incubated at 27°C with αHA mAb-coated 4.5 µm bright blue microspheres. Immediately after the addition of target beads, images were collected at 10 s intervals for ~8 min using a Zeiss LSM 710 laser scanning confocal microscope.

Video S6 Live-cell imaging of IpLITR 1.1b-mediated target interactions. Rat basophilic leukemia-2H3 cells stably co-expressing IpLITR 1.1b and LifeAct-GFP were incubated at 37°C with αHA monoclonal antibody-coated 4.5 µm microspheres. Immediately after the addition of target beads, images were collected at 10 s intervals for ~8 min using a Zeiss LSM 710 laser scanning confocal microscope. Both the brightfield-LifeAct-GFP merged views **(A)** and the LifeAct-GFP views alone **(B)** are shown. Target bead of interest is located within the box.

Video S7 Live-cell imaging of IpLITR 1.1b-mediated target interactions. Rat basophilic leukemia-2H3 cells stably co-expressing IpLITR 1.1b and LifeAct-GFP were incubated at 37°C with αHA monoclonal antibody-coated 4.5 µm microspheres. Immediately after the addition of target beads, images were collected at 10 s intervals for ~8 min using a Zeiss LSM 710 laser scanning confocal microscope. Both the brightfield-LifeAct-GFP merged views **(A)** and the LifeAct-GFP views alone **(B)** are shown. Target bead of interest is located within the box.

Video S8 Live-cell imaging of IpLITR 1.1b-mediated target interactions. Rat basophilic leukemia-2H3 cells stably co-expressing IpLITR 1.1b and LifeAct-GFP were incubated at 37°C with αHA monoclonal antibody-coated 4.5 µm microspheres. Immediately after the addition of target beads, images were collected at 10 s intervals for ~8 min using a Zeiss LSM 710 laser scanning confocal microscope. Both the brightfield-LifeAct-GFP merged views **(A)** and the LifeAct-GFP views alone **(B)** are shown. Target bead of interest is located within the box.

Video S9 Live-cell imaging LCI of IpLITR 1.1b-mediated target interactions. Rat basophilic leukemia-2H3 cells stably co-expressing IpLITR 1.1b and LifeAct-GFP were incubated at 37°C with αHA monoclonal antibody-coated 4.5 µm microspheres. Immediately after the addition of target beads, images were collected at 10 s intervals for ~8 min using a Zeiss LSM 710 laser scanning confocal microscope. Both the brightfield-LifeAct-GFP merged views **(A)** and the LifeAct-GFP views alone **(B)** are shown. Target beads of interest are located within the box.

Video S10 Live-cell imaging of IpLITR 1.1b-mediated target interactions. Rat basophilic leukemia-2H3 cells stably co-expressing IpLITR 1.1b and LifeAct-GFP were incubated at 37°C with αHA monoclonal antibody-coated 4.5 µm microspheres. Immediately after the addition of target beads, images were collected at 10 s intervals for ~8 min using a Zeiss LSM 710 laser scanning confocal microscope. Both the brightfield-LifeAct-GFP merged views **(A)** and the LifeAct-GFP views alone **(B)** are shown. Target bead of interest is located within the box.

Video S11 Live-cell imaging of IpLITR 1.1b-mediated target interactions. Rat basophilic leukemia-2H3 cells stably co-expressing IpLITR 1.1b and LifeAct-GFP were incubated at 37°C with αHA monoclonal antibody-coated 4.5 µm microspheres. Immediately after the addition of target beads, images were collected at 10 s intervals for ~8 min using a Zeiss LSM 710 laser scanning confocal microscope. Both the brightfield-LifeAct-GFP merged views **(A)** and the LifeAct-GFP views alone **(B)** are shown. Note: five target beads are interacted with by the cell over the duration of the video.

Video S12 Live-cell imaging of IpLITR 1.1b-mediated phagocytosis. Rat basophilic leukemia-2H3 cells stably co-expressing IpLITR 1.1b and LifeAct-GFP were incubated at 37°C with αHA monoclonal antibody-coated 4.5 µm bright blue microspheres. Immediately after the addition of target beads, images were collected at 10 s intervals for ~8 min using a Zeiss LSM 710 laser scanning confocal microscope. Target bead of interest is located within the box.

Video S13 Live-cell imaging of IpLITR 1.1b-mediated phagocytosis. Rat basophilic leukemia-2H3 cells stably co-expressing IpLITR 1.1b and LifeAct-GFP were incubated at 37°C with αHA monoclonal antibody-coated 4.5 µm bright blue microspheres. Immediately after the addition of target beads, images were collected at 10 s intervals for ~8 min using a Zeiss LSM 710 laser scanning confocal microscope. Target bead of interest is located within the box.

Video S14 Live-cell imaging of IpLITR 1.1b-mediated phagocytosis. Rat basophilic leukemia-2H3 cells stably co-expressing IpLITR 1.1b and LifeAct-GFP were incubated at 37°C with αHA monoclonal antibody-coated 4.5 µm bright blue microspheres. Immediately after the addition of target beads, images were collected at 10 s intervals for ~8 min using a Zeiss LSM 710 laser scanning confocal microscope. Target bead of interest is located within the box.

Video S15 Live-cell imaging of IpLITR 1.1b-mediated phagocytosis. Rat basophilic leukemia-2H3 cells stably co-expressing IpLITR 1.1b and LifeAct-GFP were incubated at 37°C with αHA monoclonal antibody-coated 4.5 µm bright blue microspheres. Immediately after the addition of target beads, images were collected at 10 s intervals for ~8 min using a Zeiss LSM 710 laser scanning confocal microscope. Target bead of interest is located within the box, which was pre-captured by another cell that is visible in the top right corner of the video.

Video S16 Live-cell imaging of IpLITR 1.1b-mediated phagocytosis at 27^o^C. Rat basophilic leukemia-2H3 cells stably co-expressing IpLITR 1.1b and LifeAct-GFP were incubated at 27°C with αHA monoclonal antibody-coated 4.5 µm bright blue microspheres. Immediately after the addition of target beads, images were collected at 10 s intervals for ~8 min using a Zeiss LSM 710 laser scanning confocal microscope. Target bead of interest is located within the box.

Video S17 Live-cell imaging of IpLITR 1.1b-mediated phagocytosis at 27°C. Rat basophilic leukemia-2H3 cells stably co-expressing IpLITR 1.1b and LifeAct-GFP were incubated at 27°C with αHA monoclonal antibody-coated 4.5 µm bright blue microspheres. Immediately after the addition of target beads, images were collected at 10 s intervals for ~8 min using a Zeiss LSM 710 laser scanning confocal microscope. Target bead of interest is located within the box.

Video S18 Live-cell imaging of IpLITR 1.1b-mediated phagocytosis at 27°C. Rat basophilic leukemia-2H3 cells stably co-expressing IpLITR 1.1b and LifeAct-GFP were incubated at 27°C with αHA monoclonal antibody-coated 4.5 µm bright blue microspheres. Immediately after the addition of target beads, images were collected at 10 s intervals for ~8 min using a Zeiss LSM 710 laser scanning confocal microscope. Target bead of interest is located within the box.

Video S19 Live-cell imaging of IpLITR 1.1b-mediated phagocytosis at 27°C. Rat basophilic leukemia-2H3 cells stably co-expressing IpLITR 1.1b and LifeAct-GFP were incubated at 27°C with αHA monoclonal antibody-coated 4.5 µm bright blue microspheres. Immediately after the addition of target beads, images were collected at 10 s intervals for ~8 min using a Zeiss LSM 710 laser scanning confocal microscope. Target bead of interest is located within the box.

Video S20 Live-cell imaging of IpLITR 1.1b-mediated phagocytosis at 27°C. Rat basophilic leukemia-2H3 cells stably co-expressing IpLITR 1.1b and LifeAct-GFP were incubated at 27°C with αHA monoclonal antibody-coated 4.5 µm bright blue microspheres. Immediately after the addition of target beads, images were collected at 10 s intervals for ~8 min using a Zeiss LSM 710 laser scanning confocal microscope. Target bead of interest is located within the box.

## Abbreviations

FcR, Fc receptor; SEM, scanning electron microscopy; Rac1/2, ras-related C3 botulinum toxin substrate ½; Cdc42, cell division control protein 42 homolog; PtdIns(3,4,5)P3, phosphatidylinositol 3,4,5-trisphosphate; IpLITRs, channel catfish *Ictalurus punctatus* leukocyte immune-type receptors; RBL-2H3, rat basophilic leukemia-2H3; ITAM, mammalian immunoreceptor tyrosine-based activation motif; ITIM, immunoreceptor tyrosine-based inhibitory motif; LCI, live-cell imaging; GFP, green fluorescent protein; HA, hemagglutinin; CYT, cytoplasmic tail; MEM, minimal essential media; mAb, monoclonal antibody; PBS, phosphate buffered saline; BB, blue beads; Nck, non-catalytic region of tyrosine kinase adaptor protein; Wave2, N-WASp family verprolin-homologous protein-2; Src, proto-oncogene tyrosine-protein kinase Src; Vav, proto-oncogene Vav; Syk, spleen tyrosine kinase; PtdSer, phosphatidylserine; PSR, PtdSer receptor.
